# The Role of Neck Circumference as a Screening Tool for Obesity in Female Adults: A Cross-Sectional Study in Western Maharashtra

**DOI:** 10.7759/cureus.65814

**Published:** 2024-07-31

**Authors:** Gayatri R Nair, Sudhir L Jadhav, Deepu Palal, Hetal Rathod, Prerna Verma, Jitendra Bhawalkar, Manisha A Rathi, Suman Ray, Divya Madamanchi

**Affiliations:** 1 Department of Community Medicine, Dr. D.Y. Patil Medical College, Hospital and Research Centre, Dr. D.Y. Patil Vidyapeeth, Pune, IND; 2 Department of Community Physiotherapy, Dr. D.Y. Patil Medical College, Hospital and Research Centre, Dr. D.Y. Patil Vidyapeeth, Pune, IND

**Keywords:** females, metabolic, screening, neck circumference, obesity

## Abstract

Introduction: The rising prevalence of obesity has become a global public health crisis. Traditional screening tools like body mass index (BMI) and waist circumference (WC) have limitations, prompting the need for simpler, more effective alternatives. Neck circumference (NC) has emerged as a promising tool due to its simplicity, affordability, and reliability. The study aimed to evaluate neck circumference as a screening tool for obesity among female adults, alongside measuring BMI, waist-to-hip ratio (WHR), WC, and NC, and establishing NC cut-off values using body fat percentage as the gold standard. Additionally, it sought to compare the predictive accuracy of these measures for assessing obesity.

Methods: This cross-sectional study, conducted from March 2023 to October 2023, involved 362 female students from a health sciences university in Western Maharashtra, India. The participants provided informed consent and underwent anthropometric measurements, including height, weight, waist circumference, hip circumference, body fat percentage, and neck circumference. Body fat percentage, measured using a bioimpedance analyzer, served as the reference standard.

Results: The study identified a neck circumference (NC) cut-off of 31.3 cm using receiver operating characteristic (ROC) analysis, showing robust sensitivity (71.23%) and specificity (79.02%) for detecting obesity defined by body fat percentage. Waist circumference (WC) showed the highest sensitivity (73.97%) for diagnosing obesity in females, followed by NC (71.23%).

Conclusion: Neck circumference is a practical, cost-effective, and reliable screening tool for obesity, offering advantages over traditional methods. Its noninvasive nature and ease of measurement make it suitable for large-scale screening, contributing to the early detection and management of obesity-related health risks. This study supports the inclusion of NC in routine clinical assessments and public health initiatives.

## Introduction

The escalating prevalence of overweight and obesity has emerged as a significant public health crisis globally [1]. According to recent reports by the World Health Organization (WHO), over 1.9 billion individuals worldwide are classified as overweight, with 650 million suffering from obesity [2]. Projections from the World Obesity Atlas 2022 indicate that by 2030, one billion people will live with obesity [3]. In India alone, the prevalence of obesity among adults stands at a concerning 40.3% [4]. Obesity is now a substantial contributor to the global disease burden and significantly heightens the risk of various cardiometabolic disorders, such as diabetes, hypertension, dyslipidemia, coronary heart disease, stroke, and certain cancers. Given its modifiable nature, early screening and prevention are paramount [5].

However, gold-standard procedures for evaluating visceral fat, such as ultrasound, computed tomography, magnetic resonance imaging and dual-energy X-ray absorptiometry, are expensive and impractical for large-scale epidemiological studies or routine screenings [6]. Common screening tools, such as body mass index (BMI), although widely used, have limitations. BMI fails to account for the distribution of body fat, body composition, age-related muscle mass changes, or differences in fat distribution between the sexes [7]. Consequently, using BMI alone may lead to inaccurate estimations of adiposity and the risk of associated metabolic issues [8]. Moreover, its computation at the community level can be challenging [9].

Waist circumference (WC), another anthropometric measurement, has long been used to assess central adiposity and demonstrates a high correlation with metabolic and cardiovascular risk [6]. However, WC measurements are prone to observer error and can be influenced by various factors such as postprandial abdominal distention and respiratory movements [10,11]. Additionally, practical difficulties, a lack of standardization, and cultural considerations hinder its widespread use [8,12].

Accurate measurement of body fat is essential for managing metabolic diseases; however, direct methods often require complex and expensive equipment. Bioelectrical impedance analysis (BIA) is commonly used and validated against reference techniques, such as total body water hydrodensitometry, dual-energy X-ray absorptiometry (DEXA), and air displacement plethysmography [13]. However, the costliness of BIA, DEXA scanning, and magnetic resonance analysis highlights the necessity for simpler, more affordable techniques to measure visceral fat. Additionally, research has shown a strong association between upper body obesity and various health conditions, a link initially recognized by Jean Vague through neck skin-fold thickness assessment [14,15]. Therefore, there is an urgent need to develop cost-effective methods to assess body fat distribution and effectively manage obesity-related health risks.

In the quest for a simple, practical, and affordable alternative, neck circumference (NC) has emerged as a promising anthropometric measure. NC, being simple, quick, inexpensive, non-invasive, and less susceptible to external factors like age or breathing patterns, holds distinct advantages over other methods [16]. Studies have shown positive correlations between NC and various cardiovascular risk factors, metabolic syndrome components, and even visceral adipose tissue [17].

Moreover, NC's low intra- and inter-observer variability makes it suitable for mass screening in larger populations, including children [11]. Despite its potential, NC is often overlooked in research and clinical assessments, warranting further investigation and inclusion in routine screenings. This study was conducted to assess the role of neck circumference as a screening tool for obesity among female adults in a tertiary care hospital in Western Maharashtra. This study sought to determine cut-off values for neck circumference to identify obesity using body fat % as the standard measured by a bioelectrical impedance analyzer. Additionally, it aimed to compare the effectiveness of body mass index, waist-hip ratio, waist circumference, and neck circumference in predicting obesity.

## Materials and methods

This cross-sectional study was conducted at the Health Sciences University in Pune from March 2023 to October 2023, following ethical clearance from the Institute’s Ethics Committee. The study population comprised female undergraduate and postgraduate students from a health sciences university. Healthy adults willing to participate were included in the study. Individuals with thyroid diseases or abnormalities, neck masses, kyphosis, scoliosis, head or neck bony deformities, pregnancy, critical illness, chronic conditions such as hypertension or metabolic disorders, and those on systemic corticosteroids were excluded from the study. Convenience sampling was used to select participants for the study.

Assuming the sensitivity of neck circumference as an anthropometric tool to predict obesity in females to be 65.6%, with an allowable error of 7% and a confidence interval of 95%, the number of obese females required for the study would be 177. Taking the prevalence as 52.6 %, the required sample size was calculated as 336 using WinPepi 11.38 software. However, we have conducted the study on 362 subjects [9].

Informed written consent was collected from all participants before conducting the study. Anthropometric measurements, including height, weight, waist circumference, hip circumference, body fat percentage, and neck circumference were obtained from the participants. The height was measured using a calibrated stadiometer. Participants stood barefoot and in minimal clothing straight against the stadiometer's backboard with heels together, toes slightly apart, and feet flat. They were instructed to keep their arms hanging loosely on their sides and to ensure that their heels, buttocks, and shoulder blades were in contact with the stadiometer. The participant's head was aligned in the "Frankfort plane," and they were asked to take a deep breath and hold it. Height was measured thrice, with the participant stepping off the stadiometer between each measurement. All three measurements needed to be within 2 mm of each other; if not, additional measurements were taken until three consecutive results were within this 2 mm range [18].

The weight of the study participants was measured without shoes and wearing only light clothes, using a bioimpedance analyzer machine (BCA -1C). Waist circumference (WC, cm) was measured using non-stretchable tape at the midpoint between the lower margin of the last palpable rib and the top of the iliac crest in the mid-axillary line, with participants in a standing position at the end of a gentle expiration. Hip circumference (HC in cm) was measured in centimeters, with a tape parallel to the floor at the widest portion of the buttocks, while the subject stood with feet placed together [19]. BMI (kg/m^2^) and WHR were calculated. Neck circumference was measured horizontally just below the larynx, perpendicular to the long axis of the neck, with the participants instructed to maintain a straight posture. During this reading, the subject was asked to look straight ahead, with shoulders down, but not hunched [20]. Height, waist circumference, head circumference, and neck circumference were measured to the nearest 0.1 cm. Three readings were obtained for each measurement, and the average of all measurements was used for the analysis.

Body fat percentage was measured using a bioimpedance analyzer (BCA-1C) after participants made sure that they had rested for 20 min, had minimal clothing, and had an empty bladder and stomach. Each participant was then asked to stand on the machine barefooted in a manner that both heels and the balls of their feet touched the foot electrode. They were instructed to hold the hand electrode in their hands and stand still for 2-3 min. The core principle of BIA is the interaction between different body tissues and electrical currents. Essentially, tissues possess unique electrical characteristics: lean tissues, containing significant water content, conduct electricity effectively, whereas bone and adipose tissues, primarily composed of dielectric materials, impede the flow of electrical currents [21].

According to the Asian BMI criteria, a BMI of less than 18.5 is considered underweight, 18.5 to 22.9 is normal, 23 to 24.9 is overweight, and a BMI ≥ 25 or greater is classified as obese. According to Indian guidelines, women with a body fat percentage of > 30% are classified as obese. The cutoff for waist circumference indicating obesity in women is ≥80 cm, and the cutoff for the waist-hip ratio indicating obesity in women is ≥0.85 [22-25].

Cohen's guidelines for interpreting Kappa values are as follows: ≤ 0 indicates no agreement, 0.01 to 0.20 indicates slight agreement, 0.21 to 0.40 represents fair agreement, 0.41 to 0.60 denotes moderate agreement, 0.61 to 0.80 signifies substantial agreement, and 0.81 to 1.00 indicates almost perfect agreement. Intraclass Correlation Coefficient, values below 0.5 indicate poor reliability, values between 0.5 and 0.75 suggest moderate reliability, values from 0.75 to 0.9 indicate good reliability, and values above 0.9 represent excellent reliability [26,27].

Collected data were entered in MS Excel, and analysis was performed using MedCalc. Categorical variables were summarized using frequencies (N) and percentages (%) with 95% confidence intervals, when applicable. Continuous variables were reported as mean and standard deviation (SD) or median and interquartile range (IQR) based on the Shapiro-Wilk test for normal distribution. A Receiver Operating Characteristic (ROC) Curve for neck circumference was used to determine the cutoff value using Youden's index. The sensitivity and specificity of BMI, waist circumference, and waist-to-hip ratio were assessed against body fat percentage. The reliability of neck circumference measurements was evaluated using the Intraclass Correlation Coefficient for three readings.

## Results

The study included participants aged 18-36 years, with a median age of 21 years (IQR: 19-23) and a mean age of 21.42 years (SD: 3.24). Of the 362 participants, 182 (50.28%) were enrolled in the physiotherapy course, and 180 (49.72%) were enrolled in the medical course.

The BMI of the participants ranged from 13.38 kg/m² to 40.74 kg/m², with a median of 22.84 kg/m² (IQR: 19.76-26.09) and a mean of 23.33 kg/m² (SD: 4.81). Waist circumference varied from 55.33 cm to 110.73 cm, with a median of 79.25 cm (IQR: 70.83-85.97) and a mean of 79.07 cm (SD: 10.93). The waist-hip ratio ranged from 0.62 to 1.06, with a mean of 0.79 (SD: 0.1) and a median of 0.79 (IQR: 0.74-0.84). Hip circumference values spanned from 72.17 cm to 136.63 cm, with a median of 98.92 cm (IQR: 92.03-105.43) and a mean of 99.48 cm (SD: 10.35). Body fat percentage among participants ranged from 10.80% to 50.00%, with a median of 32.10% (IQR: 26.30%-37.50%) and a mean of 31.86% (SD: 8.13). Table 1 presents the distribution of participants according to various obesity parameters.

**Table 1:** Distribution of participants according to various obesity parameters

1. BMI	N (%)	95 % CI
<25	237(65.47)	60.43-70.18
≥ 25	125(34.53)	29.82-39.57
2. Waist Circumference	N(%)	95 % CI
<80	185(51.10)	45.97-56.22
≥80	177(48.90)	43.78-54.03
3. Waist Hip Ratio	N(%)	95 % CI
<0.85	283(78.18)	73.64-82.12
≥0.85	79(21.82)	17.88-26.36
4. Body Fat Percentage	N(%)	95 % CI
>30%	219(60.50)	55.38-65.40
≤30%	143(39.50)	34.60-44.62
Total	362 (100)	

Table 2 shows the diagnostic accuracy of BMI, waist circumference, and waist-hip ratio in identifying individuals with a body fat percentage greater than 30%, indicating their effectiveness in screening for obesity.

**Table 2:** Diagnostic accuracy of BMI, waist circumference, and waist-hip ratio in relation to body fat percentage

	Body Fat Percentage		Total
	>30 %	≤ 30%	
1. BMI			
≥25	124(56.6%)	1(0.7%)	125(34.5%)
<25	95(43.4%)	142(99.3%)	237 (65.5%)
2. Waist Circumference			
≥80 cm	162(74.0%)	15(10.5%)	177 (48.9%)
<80 cm	57(26.0%)	128(89.5%)	185 (51.1%)
3. Waist-Hip Ratio			
≥0.85	59(26.9%)	20(14.0%)	79 (21.8%)
<0.85	160(73.1%)	123(86.0%)	283(78.2%)
Total	219(60.5%)	143(39.5%)	

**Figure 1:**
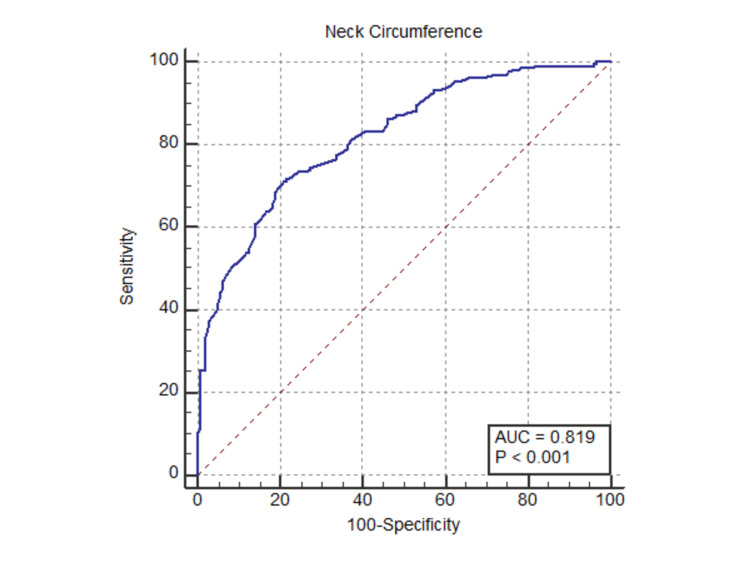
Receiver operating characteristic curve for neck circumference

Neck circumference readings of the participants ranged from 25.83 cm to 48.50 cm, with a median (IQR) of 31.33 (30.07-33.17) cm and a mean (SD) of 31.70 (2.39) cm. A receiver operating characteristic (ROC) curve was plotted to determine the optimal cutoff point for neck circumference (Figure 1).

The Area Under the ROC Curve (AUC) for neck circumference was 0.819 (95% CI: 0.78 to 0.86), indicating good diagnostic accuracy; the Z value for the AUC was 14.59, with a P value of <0.0001, demonstrating statistical significance; the optimal cut-off point for NC was determined to be >31.3 cm, corresponding to a Youden Index of 0.5. At this threshold, the sensitivity was 71.23%, correctly identifying 71.23% of individuals with a body fat percentage greater than 30%. The specificity at this threshold was 79.02%, accurately excluding 79.02% of individuals with a body fat percentage ≤ 30%. Table 3 shows the distribution of participants by the obtained neck circumference cut-off of 31.3 cm.

**Table 3:** Distribution of participants based on obtained neck circumference cut-off

Neck Circumference	N (%)	95 % CI
> 31.3 cm	186 (51.38)	46.24-56.49
≤ 31.3 cm	176 (48.62)	43.51-53.76
Total	362 (100)	

Among the total sample of 362 participants, 186 (51.38%) were classified as obese, while 176 (48.62%) were classified as non-obese based on the obtained neck circumference cut-off. The intraclass correlation coefficient between the three readings of neck circumference was 0.9240(0.9102-0.9360) and showed excellent reliability and consistency in the measurements. Table 4 compares the diagnostic accuracies of various anthropometric measures for diagnosing obesity based on body fat percentage.

**Table 4:** Comparing diagnostic accuracies of various anthropometric measures with reference to body fat percentage

Variables	Sensitivity	Specificity	Positive predictive Value	Negative predictive Value	Cohen’s Kappa Coefficient	Youden’s Index
BMI	55.62% (50.00-60.01)	99.3% (96.15-99.88)	99.2% (95.61-99.86)	59.92% (53.57-65.95)	0.50 (0.41-0.59)	0.56
Waist Circumference	73.97% (67.78-79.34)	89.51% (83.41-93.54)	91.53% (86.49- 94.8)	69.19% (62.2-75.4)	0.60 (0.50-0.70)	0.63
Waist Hip Ratio	26.94% (21.5-33.18)	86.01% (79.38-90.76)	74.68% (64.11-82.97)	43.46% (37.81-49.29)	0.11 (0.04-0.19)	0.13
Neck Circumference	71.23% (64.91-76.82)	79.02% (71.63-84.89)	83.87% (77.91-88.46)	64.2% (56.89-70.91)	0.48 (0.38-0.58)	0.5

## Discussion

Neck circumference (NC), initially explored by the Vague, is crucial for assessing upper-body fat distribution and obesity screening [15]. It correlates strongly with body fat percentage, body mass index (BMI), and visceral fat, as reported by Lyngdoh et al. Beyond conventional indices such as BMI and waist circumference (WC), NC independently predicts metabolic abnormalities, insulin resistance, and non-alcoholic fatty liver disease and is positively correlated with hyperuricemia and serum uric acid levels [28]. These associations highlight NC's potential of NC as a valuable screening tool for obesity-related health risks.

Our cross-sectional study, conducted from August 2022 to July 2024, involved apparently healthy undergraduate and postgraduate female students from a Health Sciences University in Western Maharashtra. The mean age of our participants was 21.42 (3.24) years, which aligns with findings from similar studies [29]. In our study, 34.53% of participants were classified as obese based on the Asia-Pacific BMI classification, a rate slightly higher than that reported in a similar South Indian study [9]. This variation in obesity prevalence highlights the regional differences that can influence public health strategies and interventions. Localized data such as ours are crucial for tailoring effective obesity prevention and management programs to specific regional demographics.

Our study reported a mean waist circumference (WC) of 79.07 ± 10.93 cm, similar to Hingorjo et al.'s study (78.17 ± 9.12 cm) [20] but lower than Verma et al.'s [12] (87.40 ± 13.53 cm) and Raju et al.'s [29] (80.57 ± 11.4 cm). In our study, 48.90% of the participants had a WC of ≥ 80 cm, which aligns with the findings of Qureshi et al. [14]. In contrast, Basu et al. [30] reported a lower percentage (29.91%) exceeding this threshold, while Murthy et al. [9] found a higher prevalence (70.3%) among females with a WC greater than this cutoff. In our study, the waist-to-hip ratio (WHR) ranged from 0.62 to 1.06, with a mean (SD) of 0.79 (0.07) and a median (IQR) of 0.79 (0.74-0.84), similar to Hingorjo et al.'s study which reported a mean WHR of 0.75 ± 0.055 [20]. Murthy et al. [9] found a higher mean WHR of 0.85 ± 0.03, while Qureshi et al. [14] reported an even higher mean WHR of 0.90 ± 0.06. These variations highlight the influence of population-specific factors on body fat distribution patterns, which may stem from variations in sample demographics, measurement techniques, genetic factors, or regional lifestyle factors.

Our study found a median (IQR) body fat percentage of 32.10 (26.30-37.50) %, with a mean (SD) of 31.86 (8.13%). In contrast, Verma et al. reported a lower mean body fat percentage of 28.69 ± 8.25% [12]. Murthy et al. reported a higher mean body fat percentage of 39.76 ± 4.6% compared to our findings [9]. In our study, 60.50% of the participants had a body fat percentage greater than 30%, while Verma et al. found a lower prevalence of 21.57%, exceeding this threshold [12]. These differences could be due to differences in age ranges among participants, which can influence body fat distribution.

In our study, waist circumference (WC) showed the highest sensitivity for diagnosing obesity in females at 73.97% (95% CI 67.78-79.34), followed by neck circumference (NC) at 71.23% (95% CI 64.91-76.82), BMI at 55.62% (95% CI 50-60.01), and waist-hip ratio (WHR) at 26.94% (95% CI 21.5-33.18). WHR would miss nearly three-quarters of obese individuals, whereas BMI would miss approximately half.

For specificity, BMI had the highest value at 99.3% (95% CI 96.15-99.88), followed by WC at 89.51% (95% CI 83.41-93.54), WHR at 86.01% (95% CI 79.38-90.76), and NC at 79.02% (95% CI 71.63-84.89). NC would incorrectly classify almost one-fifth of non-obese individuals as obese. BMI had the highest positive predictive value (PPV) at 99.2% (95% CI 95.61-99.86), followed by WC at 91.53% (95% CI 86.49-94.8), NC at 83.87% (95% CI 77.91-88.46), and WHR at 74.68% (95% CI 64.11-82.97). About a quarter of those identified as obese by WHR would actually be non-obese.

WC demonstrated the highest Negative Predictive Value (NPV) at 69.19% (95% CI 62.2-75.4), followed by NC at 64.2% (95% CI 56.89-70.91), BMI at 59.92% (95% CI 53.57-65.95), and WHR at 43.46% (95% CI 37.81-49.29). Over half of those identified as non-obese by WC would actually be obese, whereas, for BMI, this figure would be two-fifths. Overall, WC showed the highest diagnostic accuracy, closely followed by NC, highlighting its effectiveness in identifying individuals at risk of obesity-related complications, whereas WHR was less effective.

This study is constrained by its use of convenience sampling among female students from a single university in Western Maharashtra, limiting its broader applicability. Excluding individuals with specific health conditions, pregnancy, or systemic corticosteroid use may omit relevant subgroups. Furthermore, the absence of standardized criteria for neck circumference measurements and the limitations of bioimpedance analysis in distinguishing body tissues highlights the necessity for validation of methods such as DEXA and CT across diverse populations.

## Conclusions

Neck circumference showed good sensitivity and specificity comparable to other anthropometric measures studied. Neck circumference offers practical advantages: it is cost-effective, requires minimal manpower and training, and is feasible for widespread use. Moreover, it is non-invasive, culturally acceptable, and ensures patient comfort, remaining unaffected by factors such as respiratory status, meals, or dressing. Its excellent reliability across diverse age groups and ethnicities supports its utility in healthcare settings for early detection and management of conditions associated with excess adiposity. Furthermore, research has indicated its broader significance in mass screening initiatives aimed at improving community health outcomes.

Further research across diverse demographics is crucial to explore the utility of neck circumference, including longitudinal studies to track changes over time and assess its predictive validity alongside other measures in detecting obesity progression. Standardizing measurement protocols for neck circumference is essential to enhance reliability and consistency across studies, facilitating accurate comparisons and reproducibility of results. Considering the demonstrated sensitivity and specificity in our study, there is merit in including neck circumference as an additional screening tool in obesity guidelines and policies.
